# Commercial Oat‐Based Cosmetic Ingredients Promote Antioxidant Capacity and Reduce Inflammatory Markers in Skin Cellular Stress Models: An Insight Into Molecular Mechanisms In Vitro

**DOI:** 10.1111/jocd.71229

**Published:** 2026-10-14

**Authors:** Jiří Hanyk, Alena Rajnochová Svobodová, Zdeněk Dostál, Bohumil Zálešák, Jitka Vostálová

**Affiliations:** ^1^ Department of Medical Chemistry and Biochemistry, Faculty of Medicine and Dentistry Palacký University Olomouc Czech Republic; ^2^ Department of Plastic and Aesthetic Surgery University Hospital Olomouc Olomouc Czech Republic

**Keywords:** atopic dermatitis, human fibroblasts, human keratinocytes, NF‐κB, Nrf2, oat products, UVA protection

## Abstract

**Background:**

Dermal products containing oat ( *Avena sativa* L.) have a wide range of benefits on human skin and are commonly incorporated into skincare products.

**Aims:**

The study focused on evaluating the antioxidant, cytoprotective, and anti‐inflammatory potential of three commercial sources of oat bioactive compounds—Aura*firm* P, AvenaPLex, and Oat Lipid e.

**Methods:**

Antioxidant activity was assessed by interaction with a stable 2,2‐diphenyl‐1‐picrylhydrazyl radical and measurement of H_2_O_2_ consumed in a Fenton‐like reaction. The UVA‐protective potential was evaluated in human skin cells by measuring cell viability and reactive oxygen species (ROS) levels. The modulation of intracellular localization of nuclear factor erythroid 2‐related factor 2 (Nrf2) was investigated immunohistochemically. The anti‐inflammatory effect was evaluated in atopic dermatitis (AD)‐like models induced by tumor necrosis factor alpha and interferon gamma in vitro. Effects on nuclear factor kappa B signaling were also studied.

**Results:**

Minimal effect on UVA‐induced damage was observed on cell viability, but all three oat‐based products significantly decreased ROS production. All products stimulated Nrf2 nuclear translocation. In AD‐like models, reductions in interleukin‐6 and interleukin‐8 levels were noted. The anti‐inflammatory activity of all tested products was associated with the moderate inhibition of p50 phosphorylation.

**Conclusion:**

The studied oat‐based products have promising protective activity in skin cell stress models in vitro.

**Abbreviations**
AD: atopic dermatitis
Akt: protein kinase B
ARE: antioxidant response element
AVNs: avenanthramides
DAB: 3,3′‐diaminobenzidine
DEX: dexamethasone
DMSO: dimethyl sulfoxide
DPPH: 2,2‐diphenyl‐1‐picrylhydrazyl
HaCaT: spontaneously immortalized human keratinocyte cell line
Hek‐293: immortalized human embryonic kidney 293 cell line
HO‐1: heme oxygenase‐1
HRP: horseradish peroxidase
IFN‐γ: interferon gamma
IL: interleukin
IκB‐α: inhibitor of nuclear factor kappa B
Keap1: Kelch‐like ECH‐associated protein 1
NF‐κB: nuclear factor kappa B
NHDF: normal human dermal fibroblasts
NR: neutral red
Nrf2: nuclear factor erythroid 2‐related factor 2
p50: NF‐κB p50 subunit
p‐p50: phosphorylated p50
ROS: reactive oxygen species
SFN: sulforaphane
TNF‐α: tumor necrosis factor alpha

## 1 Introduction

The skin plays an important role in protecting the body from external stressors (e.g., radiation, allergens, irritants, microbes). However, its functionality is also influenced by a variety of internal factors. The imbalance in skin homeostasis can lead to the development of various skin disorders. Atopic dermatitis (AD) is one of the most common chronic, relapsing, inflammatory skin diseases, which occurs mainly in childhood and persists and/or recurs in adulthood. AD affects about one‐fifth of all individuals during their lifetime, but the prevalence of the disease varies greatly throughout the world [1, 2]. The mechanism of AD is still not completely understood. Changes in epidermal barrier function and skin microbiome are accompanied by Th2‐driven immune responses. Pathogenesis of AD is characterized by the upregulation of pro‐inflammatory cytokines, dry erythematous lesions and intense pruritus. The inflammatory environment of AD affects epidermal organization, differentiation and protein expression [3, 4]. Nowadays, AD treatment options include emollients, moisturizers and glucocorticoids or immunosuppressants, which are applied locally in mild and moderate forms or systemically in severe forms [5]. If corticosteroids are insufficiently or poorly tolerated, AD is treated by phototherapy (UVA or UVB light) or photochemotherapy (cotreatment with photosensitisers) [6]. Therefore, the main focus is on finding approaches that would suppress the manifestations of AD. One such option is the application of natural substances or extracts that would be well tolerated and have beneficial effects on AD‐impaired skin [7].

Oats ( *Avena sativa* L.), members of the *Poaceae* family, are regarded as a nutraceutical and natural remedy for various health conditions. Their favorable effects are related to the oat constituents, which comprise saccharides (mainly starch), proteins, lipids, fiber, vitamins, and minerals [8]. Oat lipids are primarily composed of triacylglycerols, along with unsaturated fatty acids (e.g., linoleic and linolenic acids). Oats also contain phospholipids, ceramides, glycolipids and sterols, which are important for the proper functioning of the cell membrane [9, 10]. The other active phytochemicals include β‐glucans, which constitute approximately 2%–6% of the composition. They are known for their diverse biological functions, including high water‐binding capacity, promotion of wound healing [11], and modulation of immune responses [12]. Oats are also an important source of secondary metabolites, including phenolic compounds and alkaloids [13]. Avenanthramides (AVNs), a group of polyphenolic alkaloids, are exclusively found in oats and are considered the main active components of oats. The three most predominant AVNs in oats are formed from hydroxyanthranilic acid and *p*‐coumaric (AVN A), ferulic (AVN B), or caffeic acid (AVN C). AVNs have numerous physiological effects, including anti‐inflammatory, antiproliferative and antipruritic activities, which help relieve itching in dry skin [14, 15]. They have free‐radical scavenging potency, prevent lipid peroxidation and cellular oxidative damage [15, 16]. Furthermore, they upregulate genes driven by nuclear factor NF‐E2‐related factor 2 (Nrf2) such as heme oxygenase‐1 (HO‐1) and NAD(P)H:quinone oxidoreductase 1 (NQO1). In contrast, nuclear factor kappa B (NF‐κB)‐driven pathway [17] as well as phosphatidylinositol 3‐kinase (PI3K)/protein kinase B (Akt) associated pathway are downregulated by AVNs [16]. The other substances with antioxidant properties include flavonoids and phenolic acids (e.g., caffeic, coumaric, vanillic acid) [8]. They exhibit direct antioxidant effects as well as modulating the immune defense system in a manner similar to that of AVNs, via the activation of Nrf2 and inhibition of NF‐κB [18, 19] and PI3K/Akt [20] pathways.

Several in vitro and/or in vivo studies have described the anti‐inflammatory, skin barrier repair, and moisturizing effect of oat products on the skin. Oat‐containing preparations are used to relieve itching, rash, and redness of the skin or improve the healing of burns [7, 21, 22]. Colloidal oatmeals are commonly used as adjunctive therapy for pruritic and dry skin conditions [15]. They help repair the integrity of the skin barrier and stabilize the skin microbiome [23, 24]. The above information shows oat‐based products are an attractive natural ingredient for dermal applications. This study focused on evaluating the molecular mechanisms (particularly the antioxidant, cytoprotective, and anti‐inflammatory potential) of three different commercial oat products—Aura*firm* P, AvenaPLex, and Oat Lipid e. Although these products differ in their active compound composition, practical user experience provides evidence of the beneficial effects of formulations containing these oat ingredients. Cosmetic companies utilize these specific oat derivatives at percentage concentrations to manufacture dermatological preparations. Oat Lipid e (oat oil) is used in formulations for restoring the barrier function (anti‐aging) at concentrations of 3%–5% and in preparations for people with AD at concentrations of 5%–15%. Aura*firm* P (fermented colloidal oatmeal) is used in preparations stabilizing skin microflora and balancing pH at concentrations of 1%–2%. AvenaPLex (crude oat kernel extract) is an active ingredient in regenerative cosmetics for severely damaged skin at concentrations of 1%–1.5%. For the detailed composition of the individual oat products, see Table S1.

## 2 Materials and Methods

### 2.1 Chemicals and Reagents

The three commercial products Aura*firm* P, AvenaPLex, and Oat Lipid e were provided by the company Oat Services Ltd. (Hampshire, UK). Chemical characterization is shown in Table S1. The specifications for the rest of the chemicals and reagents used are provided in the Supporting Information.

### 2.2 Cell Cultures

Normal human dermal fibroblasts (NHDF) were isolated from a donor woman's breast skin fragments. The superfluous skin was obtained from healthy donors undergoing plastic surgery at the Department of Plastic and Aesthetic Surgery. The use of skin tissue complied with the Ethics Committee. All volunteers had signed their written informed consent. The skin was transported in phosphate‐buffered solution (PBS) with a mixture of antibiotics (penicillin (0.5 mg/mL), streptomycin (500 U/mL), and amphotericin B (1.25 μg/mL)). The tissue was cut into pieces (1 × 1 cm), placed in Petri dishes, and cultivated as described by Ryšavá et al. [25]. NHDF were grown in culture medium (DMEM supplemented with fetal bovine serum (FBS; 10%; *v*/*v*), penicillin (0.1 mg/mL), and streptomycin (100 U/mL)), and incubated in a humidified atmosphere with CO_2_ (5%; *v/v*) at 37°C. For experiments, NHDF were used in the 2nd‐4th passage and seeded onto 96‐well plates at a density of 0.5 × 10^5^ cells/cm^2^ in the culture medium and allowed to attach overnight to reach confluence.

A spontaneously immortalized human keratinocyte cell line (HaCaT) was purchased from CLS Cell Lines Service (Eppelheim, Germany). The HaCaT cells were cultured in the same culture medium and under the same conditions as the fibroblasts (see above). For experiments, HaCaT cells were used in 50th–60th passages and seeded onto 96‐well or 6‐well plates or 6‐cm Petri dishes at a density of 1.0 × 10^5^ cells/cm^2^ in the culture medium and allowed to attach overnight to reach confluence.

The human embryonic kidney 293 cell line (Hek‐293) was obtained from ECACC (No. 85120602) via Sigma‐Aldrich as the provider (St. Louis, MO, USA). The Hek‐293 cells were cultured in DMEM supplemented with FBS (10%; *v*/*v*), sodium pyruvate (1 mmol/L), penicillin (0.1 mg/mL), and streptomycin (100 U/mL). The cultivation conditions were the same as for NHDF and HaCaT. For experiments, Hek‐293 cells were used in 10th‐15th passages and seeded onto 96‐well plates at a density of 0.5 × 10^5^ cells/cm^2^ in the culture medium and allowed to attach overnight to reach 80% confluence.

### 2.3 Evaluation of Cytotoxicity

Stock solutions of oat products (0.1–1.0 g/mL; DMSO) were diluted in serum‐free medium to obtain the final concentrations of 0.6–5.0 mg/mL. The range was chosen based on solubility limitations at higher concentrations of the products. The solutions were applied to cells (HaCaT or NHDF) for 24 and 48 h. Serum‐free medium with DMSO (0.5%; *v/v*) was used as a negative control. The viability of cells was determined by neutral red (NR) uptake assay. Briefly, after the treatment period, medium with oat products was removed and NR solution (0.02%; w/v) in PBS with glucose (PBS‐G; 1 mg/mL; 50 μL) was applied for 1.5 h at 37°C. Then, NR solution was discarded and cells were fixed with a mixture of formaldehyde (0.25%; *v/v*) and CaCl_2_ (0.5%; *w*/*v*). NR accumulated in cells was dissolved in acetic acid (1%; *v/v*) in methanol (50%; *v/v*). The plates were shaken for 20 min, and absorbance was measured at 540 nm using a microplate reader (Infinite 200 PRO, Tecan, Austria).

### 2.4 Photoprotective and Regenerative Effect

The photoprotective (pretreatment) and regenerative (posttreatment) effect of the studied oat products were evaluated on HaCaT and NHDF cells. For photoprotection, cells on 96‐well plates (~100% confluence) were washed with PBS, and serum‐free medium with oat products (0.6–5.0 mg/mL) was applied for 1 h. Two plates were prepared in parallel. Then, the medium was discarded, cells were washed twice with PBS, and then PBS‐G (0.2 mL) was applied. One of two identical plates was subsequently exposed to UVA radiation (+UVA). HaCaT and NHDF were exposed to a phototoxic dose of 10 J/cm^2^ and 7.5 J/cm^2^, respectively. During the irradiation, the plates were placed on cold panels to eliminate the heat induced by UV radiation. A solar simulator SOL 500 (Dr. Hoenle UV Technology, Germany) equipped with a H1 filter transmitting wavelengths of 320–400 nm was used as the source of UVA radiation. The UVA output before each experiment was measured with a UVA‐meter (Dr. Hoenle UV Technology, Germany). The second plate (nonirradiated cells; ‐UVA) was kept in the dark (in the incubator) during UVA exposure. After irradiation, PBS‐G was replaced with serum‐free medium in both plates, and cells were incubated for 24 h in the incubator with CO_2_ (5%; *v/v*) at 37°C.

To study the effect on cell regeneration, cells near to confluence were washed twice with PBS, and PBS‐G was applied. In parallel, two plates were prepared. One plate was exposed to UVA radiation (+UVA). The same doses as for pretreatment were used. The second plate was kept in the dark during the irradiation period (‐UVA). After irradiation, PBS‐G on both plates was discarded and oat products (0.6–5.0 mg/mL) in serum‐free culture medium were applied for 24 h. The plates were kept under the same incubation conditions. The effects of the test products on cell viability in both pretreatment and posttreatment were evaluated by NR uptake.

### 2.5 Radical Scavenging Activity

The antioxidant activity of oat products was evaluated by 2,2‐diphenyl‐1‐picrylhydrazyl (DPPH) assay. The oat products were dissolved in methanol. The reaction mixture contained 50 μL of oat product solution and 150 μL of freshly prepared DPPH solution (20 mg/L) in methanol. Tocopherol was used as a reference compound. After 30 min of incubation, the absorbance was measured at 517 nm using a microplate reader. The radical scavenging capacity (SC) was expressed as the concentration of studied product (μg/mL) at which the absorbance of DPPH was reduced to 50% (SC_50_).

### 2.6 Antioxidant Capacity Using Fenton‐Like Reaction in Cells

Determination of antioxidant capacity in an aqueous environment was performed by indirect measurement of H_2_O_2_ consumed in a Fenton‐like reaction based on an oxidation–reduction reaction of menadione with H_2_O_2_ and the tested oat products. The Fenton‐like reaction itself lasted for 1 h. To measure residual H_2_O_2_, Hek‐293 cells transfected with the genetically encoded probe Hyper7 were used [26]. The cells were seeded onto cover slips (30 mm in diameter) and cultured for 24 h under standard conditions. Cells were then transfected with plasmid‐PolyJet mixture containing 1 μg: 3.0 μL: 100 μL, DNA: PolyJet: DMEM per well and incubated for 24 h. At the beginning of the perfusion experiment, cells were placed in a perfusion chamber PC30 (Next Generation Fluorescence Imaging/NGFI, Graz, Austria) and perfused for 1.5 min with HEPES buffer (containing HEPES (10 mmol/L), NaCl (138 mmol/L), KCl (5 mmol/L), glucose (10 mmol/L), CaCl_2_ (2 mmol/L), MgCl_2_ (1 mmol/L); pH = 7.4). Then the buffer was changed to HEPES buffer containing reaction mixture: H_2_O_2_ (10 μmol/L), menadione (10 μmol/L) and oat products in selected concentrations (0.6–2.5 mg/mL) or quercetin (25 μmol/L = 8.5 μg/mL; a positive control). The flow rate of the reservoir was manually adjusted to 1 mL/min. Olympus IX83 with a Hamamatsu Orca‐Flash 4.0 LT3 (20× objective, Olympus, Tokyo, Japan) equipped with a perfusion system was used. For excitation, two LEDs were used at 402 and 490 nm, with emission collected at 528 nm. The Hyper7 fluorescence intensity was measured over time using cellSens Dimension software, the image acquisition frequency was every 1 s. Background subtraction, using a background ROI, and photobleaching correction, using an extrapolation of the initial bleaching curve under basal conditions, was done using an excel macro. All the experiments were done at room temperature (RT) without specific temperature or gas control. Analyzed cells were chosen randomly based on the expression of the genetically encoded H_2_O_2_ biosensor. For each measured field, one well was used. Each field was chosen at random. For each dataset, at least three fields were processed (at least three independent experiments) and the average normalized intensity was plotted.

### 2.7 Effect on UVA‐Induced Intracellular ROS Production

UVA‐induced ROS production and the radical scavenging effect of the tested oat products in living cells were measured by dichlorodihydrofluorescein diacetate (H_2_DCFDA) assay. To study preventive effect, HaCaT cells on 6‐well plates were washed with PBS and serum‐free medium with oat products (0.6–5.0 mg/mL) was applied for 1 h. Then cells were washed twice with PBS and exposed to a phototoxic dose of 7.5 J/cm^2^ in PBS‐G (1.5 mL) (details see above). After irradiation, serum‐free medium was applied and cells were incubated for 2 h in the incubator. To evaluate regenerative effect, cells were washed with PBS and exposed to UVA radiation (7.5 J/cm^2^) in PBS‐G. After exposure, PBS‐G was discarded and serum‐free medium with oat products (0.6–5.0 mg/mL) was applied for 2 h. For evaluation of the intracellular ROS level, pretreated or posttreated cells were incubated with H_2_DCFDA (5 μmol/L; 20 min; incubator) in serum‐free medium. Then the cells were prepared for microscopic analysis. First, they were rinsed with PBS and then placed into HEPES buffer (see above). Microscopic evaluation was performed by using an Olympus IX83 inverted fluorescence microscope (4× objective, Tokyo, Japan). For excitation, SPECTRA X light engine (Lumencor, Beaverton, OR, USA) with a 482/520 nm ex/em filter block was used. For each sample, four fields were randomly chosen and the microphotographs were taken. The data were expressed as the average normalized fluorescence intensity assessed by ImageJ software. Representative microphotographs are presented.

### 2.8 Nrf2 Intracellular Localization

The HaCaT cells were seeded onto 8‐well chamber slides at a density of 8000 cells/well in the culture medium and allowed to attach overnight (~70% confluence). Cells were treated with oat products (0.6 and 5.0 mg/mL) or sulforaphane (SFN; 5 μmol/L; ~0.89 μg/mL; a reference substance) in serum‐free medium for 1 or 3 h. Cells were then fixed in a cold methanol: acetone mixture (1:1) for 15 min at RT, and stored at −80°C till use. Before immunocytochemical (ICC) staining, the cells were rehydrated, and heat‐induced antigen retrieval was performed in citric buffer pH 6.0 (15 min; 120°C; Histos Milestone apparatus). According to the ICC protocol, slides with cells were incubated with PolyDetector Peroxidase Blocker (15 min), Protein Block (10 min), and then with primary anti‐Nrf2 mouse monoclonal IgG diluted in antibody diluent (1:200) for 1.5 h. Subsequently, cells were incubated with the secondary antibody labeled with horseradish peroxidase (HRP) (30 min) and visualization was performed by using the substrate 3,3′‐diaminobenzidine (DAB) chromogen (5–10 min). Wash Buffer was used for washing between individual steps. Cell nuclei were counterstained with hematoxylin. Finally, samples were dehydrated and coverslipped. The assessment of intracellular Nrf2 localization was based on visual analysis of the cytoplasm and nucleus. The semi‐quantitative evaluation was performed in 3 different fields of vision at 100× magnification with an Olympus BX40 light microscope (Tokyo, Japan). Semi‐quantitative staining intensity analysis was performed by dividing the cells into four categories: negative (C1), weakly positive (C2), moderately positive (C3), and strongly positive (C4), using the software QuPath [27]. Approximately 100 cells were evaluated in 3 microscopic photographs (fields of view). The histoscore (H‐score; range 0–300) was calculated according to the formula:

$$H-\text{score}=\left(0\times \%\text{of}\;C1\right)+\left(1\times \%\text{of}\;C2\right)+\left(2\times \%\text{of}\;C3\right)+\left(3\times \%\text{of}\;C4\right)$$

### 2.9 Effects on Atopic Dermatitis (AD)‐Like Conditions

AD‐like models were set up by the application of tumor necrosis factor alpha (TNF‐α) and interferon gamma (IFN‐γ) (each 10 ng/mL) according to a previous study [28] with necessary modifications. In the pretreatment AD‐like model, HaCaT cells were preincubated with oat products (0.6 and 5.0 mg/mL) in serum‐free medium for 1 h, and then AD‐inducing agents were applied for 24 h. In the posttreatment AD‐like model, cells were first incubated with AD‐inducing agents for 24 h, and then the studied oat products (0.6 and 5.0 mg/mL) were applied for a further 24 h. Dexamethasone (DEX; 1 μmol/L; ~0.39 μg/mL) was used as a reference compound for evaluating oat product effectiveness. In both models, the medium was collected for evaluation of interleukins (ILs) levels and cell viability was determined by NR uptake (see above).

### 2.10 Determination of Interleukins

The level of IL‐6 and IL‐8 was measured using ELISA kits according to the manufacturer's instructions. Briefly, an ELISA plate (96‐wells) was coated with capture antibodies and incubated overnight at RT. Between individual subsequent steps, the plate was washed with wash buffer (Tween‐20 (0.05%; *v/v*) in PBS) four times. The subsequent steps included a blocking step with block buffer (bovine serum albumin (BSA); 1%; w/v in PBS) for at least 1 h; incubation with samples and standards (0–1.5 ng/mL) for 2 h; the application of detection antibody conjugated with biotin for 2 h; incubation with avidin‐HRP conjugate for 30 min; and the application of 2,2′‐azino‐bis(3‐ethylbenzothiazoline‐6‐sulfonic acid) substrate solution and measurement of the colored product at 450 nm with wavelength correction set at 650 nm using a microplate reader (Infinite 200 PRO, Tecan, Austria).

### 2.11 NF‐κB Signaling Pathway

Hek‐293 cells were transfected with the bi‐directional NF‐κB reporter plasmid pSGNluc [29]. Cells were transfected using Lipofectamine 3000 Reagent according to a modified version of the manufacturer's protocol. Briefly, in one test tube 0.3 μL of Lipofectamine 3000 Reagent was diluted in 5 μL of antibiotic‐ and serum‐free DMEM; in the second test tube, plasmid DNA (0.05 μg) and P3000 Reagent (0.1 μL) were diluted in 5 μL of antibiotic‐ and serum‐free DMEM; both are given for one well of a 96‐well plate. After mixing the contents of both test tubes, the transfection mixture was incubated for 10 min at RT to allow the formation of transfection complexes. Finally, the whole mixture (~10.4 μL) was applied into each well of the plate.

After incubation for 24 h, the medium was replaced with serum‐free medium with oat products (0.6 and 5.0 mg/mL), DEX (1 μmol/L; ~0.39 μg/mL; a reference compound) or MG‐132 (1 μmol/L; an inhibitor of NF‐κB inhibitor (IκB) degradation) for 1 h. Then, inflammation (the activation of NF‐κB transcription factor) was induced with TNF‐α and IFN‐γ (each 10 ng/mL). After 24 h, the enhanced green fluorescent protein (EGFP) expression level was assessed by using a Zeiss AxiovertC epifluorescent microscope (2.5× objective) with a Zeiss AxioCam ICM1 with a 470 nm/515 nm Ex/Em filter block for green fluorescence. Intensity was measured in ImageJ after background subtraction. One well was used for each measured field. Two fields per repeat were processed, and the normalized intensity was calculated (ImageJ).

### 2.12 NF‐κB Signaling Pathway‐Related Proteins

The level of selected NF‐κB signaling pathway‐related proteins (phosphorylated‐NF‐κB p50 (p‐p50) and IκB‐α) was evaluated by Western blotting as described by Ryšavá et al. [25] with necessary modifications. HaCaT cells were preincubated with oat products (0.6 and 5.0 mg/mL) in serum‐free medium for 1 h, and then AD‐inducing agents (TNF‐α and IFN‐γ; each 10 ng/mL) were applied for 30 min. DEX (1 μmol/L; ~0.39 μg/mL) was used as a reference compound for evaluating oat product effectiveness. After the experiment, cells were washed and scraped into cold PBS (with 1 mmol/L Na_3_VO_4_, 10 mmol/L NaF, 1 mmol/L phenylmethanesulfonyl fluoride) and centrifuged (5 min, 1300 rpm, 4°C). Cell pellets were resuspended in ice‐cold lysis buffer (20 mmol/L Tris, 5 mmol/L EGTA, 150 mmol/L NaCl, 20 mmol/L glycerol phosphate, 1 mmol/L NaF, 1 mmol/L Na_3_VO_4_, Triton X‐100 (1%; v/v), Tween 20 (0.1%; v/v), pH 7.5; 1 protease inhibitor cocktail tablet cOmplete per 50 mL) and total cellular lysates were centrifuged (10 min, 12 000 rpm, 4°C). The amount of proteins was determined using a BCA Protein Assay Kit.

Proteins were separated in 10% SDS‐PAGE gels and transferred to PVDF membranes (Immobilon‐P, Merck, CZ). Membranes were blocked in BSA (5%; *w/v*) in Tris‐buffered saline (TBST; 100 mmol/L Tris, NaCl (0.9%; *w/v*) with Tween‐20 (0.05%; *v/v*)) for 2 h at RT, followed by incubation with specific primary antibodies (overnight, 4°C): p‐p50 (1:500, in enhancer solution 1), IκB‐α, and β‐actin (1:500, in BSA (5%, *w/v*) in TBST). Then, membranes were washed in TBST and probed with HRP‐conjugated secondary antibody (1:5000) for 1 h at RT. After washing in TBST, proteins were visualized by chemiluminescence reagent and detected with the Alliance Q9 Advanced imaging system (UVITEC Cambridge, UK). Quantification of the protein was performed by a densitometric analysis using the NineAlliance software. The target protein expression levels were normalized to a reference protein β‐actin.

### 2.13 Direct Detection of Akt Activity

For determination of Akt activity, Hek‐293 cells transfected with the genetically encoded probe ExRai‐AktAR2 were used [30]. The cells were seeded onto coverslips (30 mm in diameter) and cultured for 24 h under standard conditions. Cells were then transfected with plasmid‐PolyJet mixture containing 1 μg: 3.0 μL: 100 μL, DNA: PolyJet: DMEM per well and incubated for 24 h. At the beginning of the perfusion experiment, cells were placed in a perfusion chamber PC30 (Next Generation Fluorescence Imaging/NGFI, Graz, Austria) and perfused for 1.0 min with HEPES buffer (see above). Then the buffer was changed to HEPES buffer containing selected oat products with final concentration 0.6 mg/mL. The flow rate of the reservoir was manually adjusted to 1 mL/min. Olympus IX83 with a Hamamatsu Orca‐Flash 4.0 LT3 (40× immersion objective, Olympus, Tokyo, Japan) equipped with a perfusion system was used. For excitation, two LEDs were used at 402 and 490 nm, with emission collected at 528 nm. The ExRai‐AktAR2 fluorescence intensity was measured over time using cellSens Dimension software; the image acquisition frequency was every 3 s. Background subtraction, using a background ROI, and photobleaching correction, using an extrapolation of the initial bleaching curve under basal conditions, were done using an excel macro. All the experiments were done at RT without specific temperature or gas control. Analyzed cells were chosen randomly based on the expression of the genetically encoded Akt biosensor. For each measured field, one well was used. Each field was chosen at random. For each dataset, three fields were processed (three independent experiments), and the average normalized intensity was plotted.

### 2.14 Statistical Analysis

Experiments on HaCaT and Hek‐293 were performed in three independent examinations. Due to the biological variability of NHDF, experiments were performed in six independent repetitions (NHDF isolated from the skin of six donors). Data are presented as the mean ± standard deviation (SD). Statistical significance was performed with an unpaired Student's *t*‐test for comparisons between two groups or a one‐way ANOVA with Dunnett's multiple comparison test on data normalized to the controls. All statistical analyses were performed using GraphPad Prism 8. Comparison between the control cells and cells treated with oat products and/or in combination with UVA or AD‐inducing agents was done. Statistical significance evaluation was performed at the 0.05, 0.02, or 0.001 level.

## 3 Results

### 3.1 Cell Viability

The cell viability of HaCaT was not reduced by any of the studied products after 24 or 48 h of incubation. The results showed a dose‐dependent increase in cell viability for AvenaPLex after 48 h of incubation (Figure S1). In contrast, AvenaPLex at the highest concentration used significantly decreased the viability of NHDF after 24 h after application. However, the decrease was nonsignificant after 48 h (Figure S1B). High SD was observed at both times (24 and 48 h) at the higher concentrations used, which most probably reflects the biological variability of NHDF from different donors.

### 3.2 UVA‐Photoprotective and Regenerative Effect

The effect of oat product pretreatment (1 h) on cell viability was evaluated on HaCaT and NHDF cells exposed to a phototoxic UVA dose of 10 and 7.5 J/cm^2^, respectively. The doses were chosen according to our previous study on the same cell types [25]. The results confirmed the phototoxic effect of UVA radiation (+UVA) compared to a nonirradiated (‐UVA) control, as cell viability was decreased by ~30% in both cell types (Figure 1A,B). Generally, the UVA protective effect of studied products was low, resulting in only a slight mitigation of the decrease in cell viability. However, a mild UVA protection of Aura*firm* P and AvenaPLex was found on HaCaT cells (Figure 1A). Aura*firm* P had a concentration‐dependent effect; AvenaPLex was the most effective at a concentration of 2.5 mg/mL. Oat Lipid e increased cell viability in irradiated NHDF as well as HaCaT, and its effect was concentration dependent, but nonsignificant. However, in the pretreatment (for 1 h) protocol, AvenaPLex and Oat Lipid e significantly increased the viability of nonirradiated cells (‐UVA) (Figure 1A).

**FIGURE 1:**
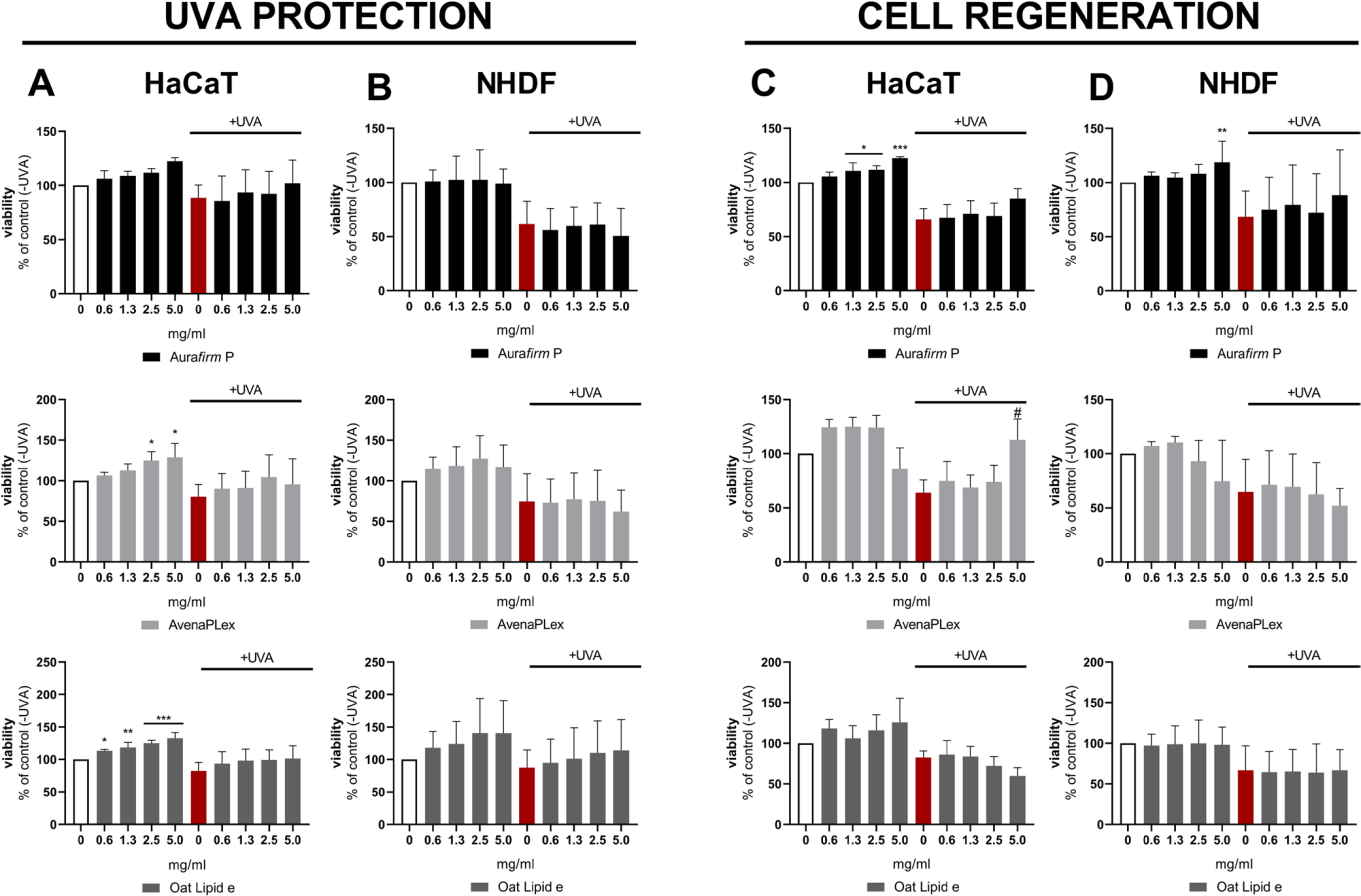
Photoprotective and regenerative effect of oat products on UVA‐irradiated cells. For UVA protection, HaCaT cells (A) and NHDF (B) were pretreated with the oat products (0.6–5.0 mg/mL) for 1 h, exposed to UVA irradiation (10 and 7.5 J/cm^2^, respectively), and then incubated with serum‐free medium for 24 h. For cell regeneration, HaCaT cells (C) and NHDF (D) were exposed to UVA irradiation, and then incubated with oat products (0.6–5.0 mg/mL) for 24 h. Cells treated with medium with DMSO (0.5%; *v/v*) were used as control cells (‐UVA; 100%). Cell viability was measured by NR uptake and data expressed as the mean ± SD (*n* = 3 for HaCaT; *n* = 6 for NHDF). * *p* ≤ 0.05, **p ≤ 0.02 or ***p ≤ 0.001 vs. control cells; #*p* ≤ 0.05 vs. UVA‐irradiated cells (+ UVA).

The regeneration effect (application of the oat products on UVA‐exposed cells for 24 h) on cell viability is shown in Figure 1C,D. Aura*firm* P increased the viability of both nonirradiated HaCaT and NHDF in a dose‐dependent manner. The observed changes were statistically significant at the highest concentration in NHDF and for 1.3–5.0 mg/mL in HaCaT. AvenaPLex partially stimulated the viability of nonirradiated cells, especially of HaCaT. However, at the highest concentration (5.0 mg/mL) it decreased the viability in both cell types (24 h application). Regardless, the most significant regenerative effect was observed at the highest concentration of AvenaPLex and reached the viability of nonirradiated HaCaT. The same treatment on NHDF had no regenerative effect (a dose‐dependent decline in viability was detected). The product Oat Lipid e moderately increased the viability of nonirradiated HaCaT, but no regeneration was found for both irradiated cells.

### 3.3 Radical Scavenging Potential

The direct antioxidant potential of the oat products was further evaluated in a cell‐free model (test tube assay) using the stable DPPH radical. Tocopherol, a known strong antioxidant [31], was used as a reference compound. Oat products exhibited a poor scavenging ability in methanol compared to tocopherol at the same concentration (312.5 μg/mL). The antioxidant effect of oat products decreased in the order AvenaPLex >>> Oat Lipid e > Aura*firm* P (Figure 2A). The radical scavenging activity was also expressed as SC_50_. Tocopherol, being a powerful antioxidant (SC_50_ = 24 ± 1.3 μmol/L; ~10.3 ± 0.6 μg/mL), exhibited a much more potent antioxidant activity than AvenaPLex (SC_50_ = 344.1 ± 67.8 μg/mL) and Oat Lipid e (SC_50_ = 4916.0 ± 745.3 μg/mL). The limited solubility of Aura*firm* P in methanol made it impossible to determine its SC_50_ (Figure 2B).

**FIGURE 2:**
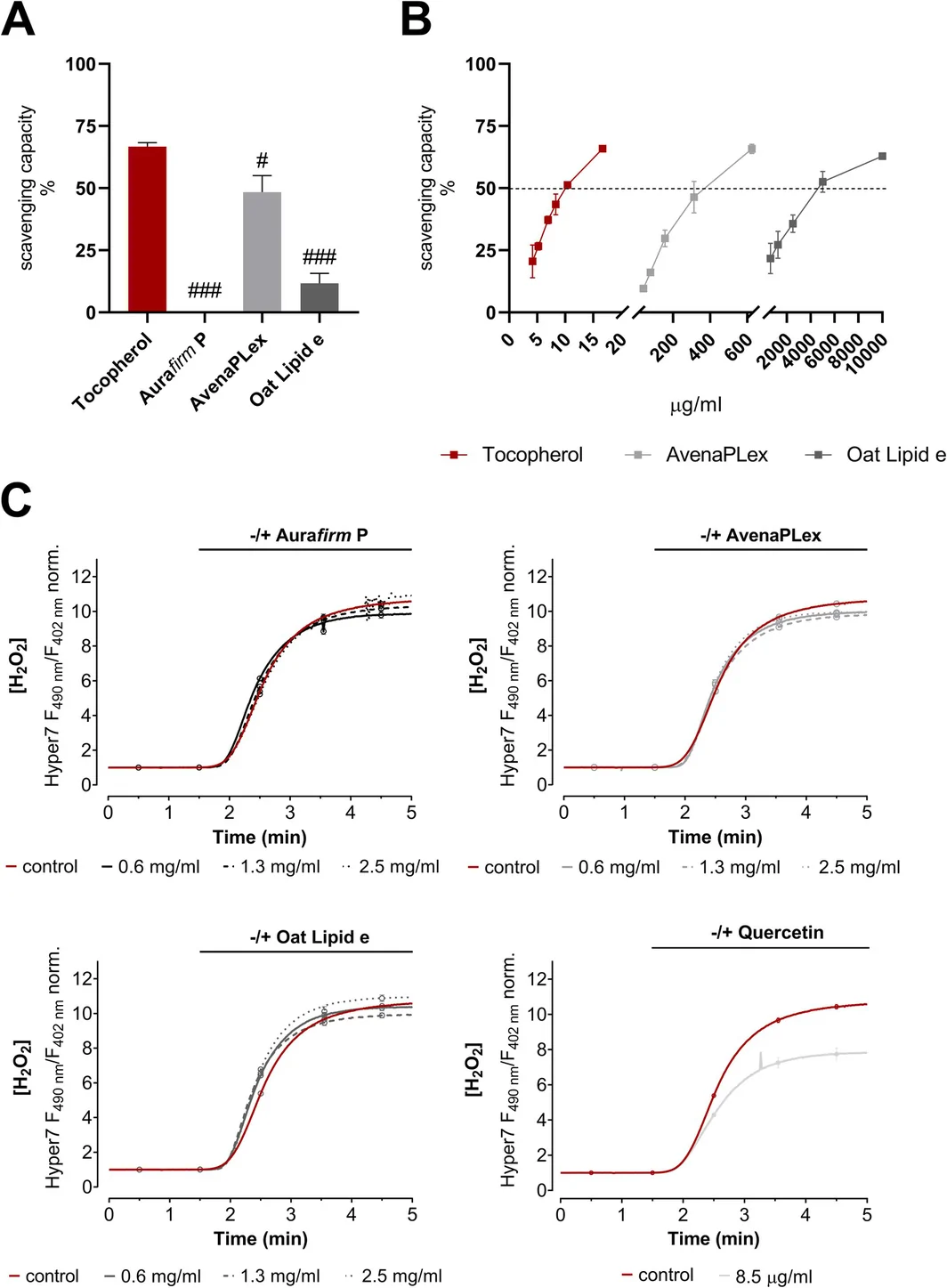
Radical scavenging and antioxidant capacity of oat products. (A) DPPH scavenging capacity was evaluated at a concentration of 312.5 μg/mL. (B) DPPH scavenging capacity expressed as SC_50_ value. Data are expressed as mean ± SD (*n* = 3). #*p* ≤ 0.05 or ### *p* ≤ 0.001 vs. tocopherol. (C) Antioxidant capacity of oat products (0.6–2.5 mg/mL) and quercetin (8.5 μg/mL) using Fenton‐like reaction. To measure residual H_2_O_2_, Hek‐293 cells transfected with the genetically encoded probe Hyper7 were used. For each dataset, at least three independent experiments were processed (*n* = 3), and the average normalized intensity (mean ± SEM) was plotted. The total number of analyzed cells was as follows: for Aura*firm* P: Control—101 cells; concentration of 0.6 mg/mL—70 cells; concentration of 1.3 mg/mL—69 cells; concentration of 2.5 mg/mL—47 cells; for AvenaPLex: Control—101 cells; concentration of 0.6 mg/mL—68 cells; concentration of 1.3 mg/mL—62 cells; concentration of 2.5 mg/mL—47 cells; for Oat Lipid e: Control—101 cells; concentration of 0.6 mg/mL—69 cells; concentration of 1.3 mg/mL—65 cells; concentration of 2.5 mg/mL—52 cells; for quercetin: Control—101 cells; quercetin—44 cells.

### 3.4 Antioxidant Capacity Using Fenton‐Like Reaction in Cells

The antioxidant properties in the aqueous environment of the studied products were evaluated by indirect measurement of H_2_O_2_ consumed in a Fenton‐like reaction based on an oxidation–reduction reaction of menadione with H_2_O_2_. The antioxidant potential of oat products (0.6–2.5 mg/mL) was compared with a known water‐soluble antioxidant quercetin (25 μmol/L; ~8.5 μg/mL). Aura*firm* P was most effective at the lowest concentration tested (0.6 mg/mL). In case of AvenaPLex, all concentrations were effective; however, the most pronounced potential was found at a concentration of 1.3 mg/mL. Oat Lipid e declined the H_2_O_2_ level only at a concentration of 1.3 mg/mL. The effect of all products was obviously lower (reduction to ~92%–93% of control) than that of quercetin (reduction to ~71% of control), see Figure 2C.

### 3.5 Effect on UVA‐Induced Intracellular ROS Production

Because oat‐based products had low efficacy in eliminating ROS generated in noncellular systems, their potential to counteract ROS produced directly in living cells by UVA radiation was further investigated. The UVA irradiation of HaCaT cells induced intensive ROS production compared to nonirradiated control cells (‐UVA) (see Figure 3A,B). The pretreatment and posttreatment by all three tested oat products significantly decreased ROS production at all used concentrations. Pretreatment with AvenaPLex showed a concentration‐dependent ROS scavenging effect (Figure 3A). Oat Lipid e was the most effective in both posttreatment and pretreatment applications (Figure 3A,B).

**FIGURE 3:**
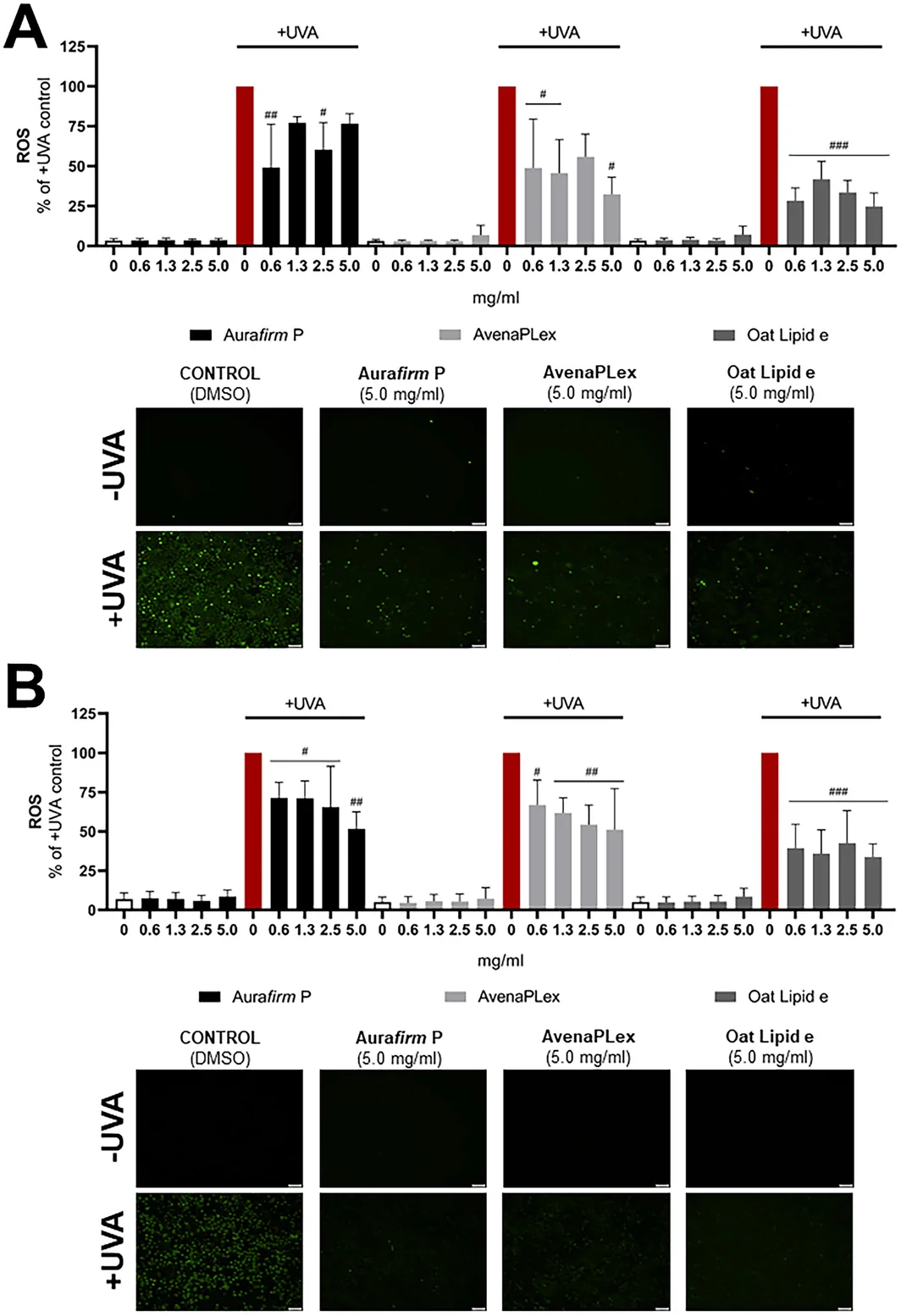
Effect of oat products on ROS level in UVA‐irradiated cells. (A) For photoprotection, HaCaT cells were incubated with the oat products (0.6–5.0 mg/mL) for 1 h, exposed to UVA irradiation (7.5 J/cm^2^), and then incubated with serum‐free medium for 2 h. (B) For cell regeneration, HaCaT cells were exposed to UVA irradiation and then incubated with oat products (0.6–5.0 mg/mL) for 2 h. Cells treated with medium with DMSO (0.5%; *v/v*) and exposed to UVA irradiation were used as a positive control (+UVA; red column; 100%). ROS amount was measured by dichlorodihydrofluorescein diacetate (H_2_DCFDA) assay. ROS level was expressed as the mean ± SD (*n* = 3). Representative images are presented. The microphotographs were taken at 20× magnification. Scale bar represents 100 μm. *p ≤ 0.05, **p ≤ 0.02 or ***p ≤ 0.001 vs. UVA‐irradiated cells (+ UVA).

### 3.6 Effect on Nrf2 Intracellular Localization

Indirect antioxidant effect of compounds is based on activation of endogenous cellular defense mechanisms, primarily through the Keap1/Nrf2/ARE signaling pathway. Its activation results in induction of endogenous antioxidant protein synthesis. Therefore, the ability of oat products to initiate one of the first steps of the pathway by transferring the Nrf2 protein from the cytoplasm to the nucleus [25] was assessed. After 1 h of treatment, all studied oat products stimulated Nrf2 nuclear translocation in HaCaT in a dose‐dependent manner. The effect is documented by the representative images and Nrf2 level in the nucleus and cytosol (Figure 4 and Figure S2). At the lower concentration (0.6 mg/mL) used, the effectiveness was in the order: AvenaPLex > Oat Lipid e > Aura*firm* P, and at the higher concentration (5.0 mg/mL) the order was: Oat Lipid e > AvenaPLex > Aura*firm* P. The effect of Oat Lipid e and AvenaPLex (5.0 mg/mL) was comparable to that of SFN, a well‐known Nrf2 activator [25]. After 3 h, all the studied oat products at both used concentrations stimulated Nrf2 translocation as well. Their effect was lower than that of SFN, except for Aura*firm* P (5.0 mg/mL), whose effect was slightly higher than that of SFN.

**FIGURE 4:**
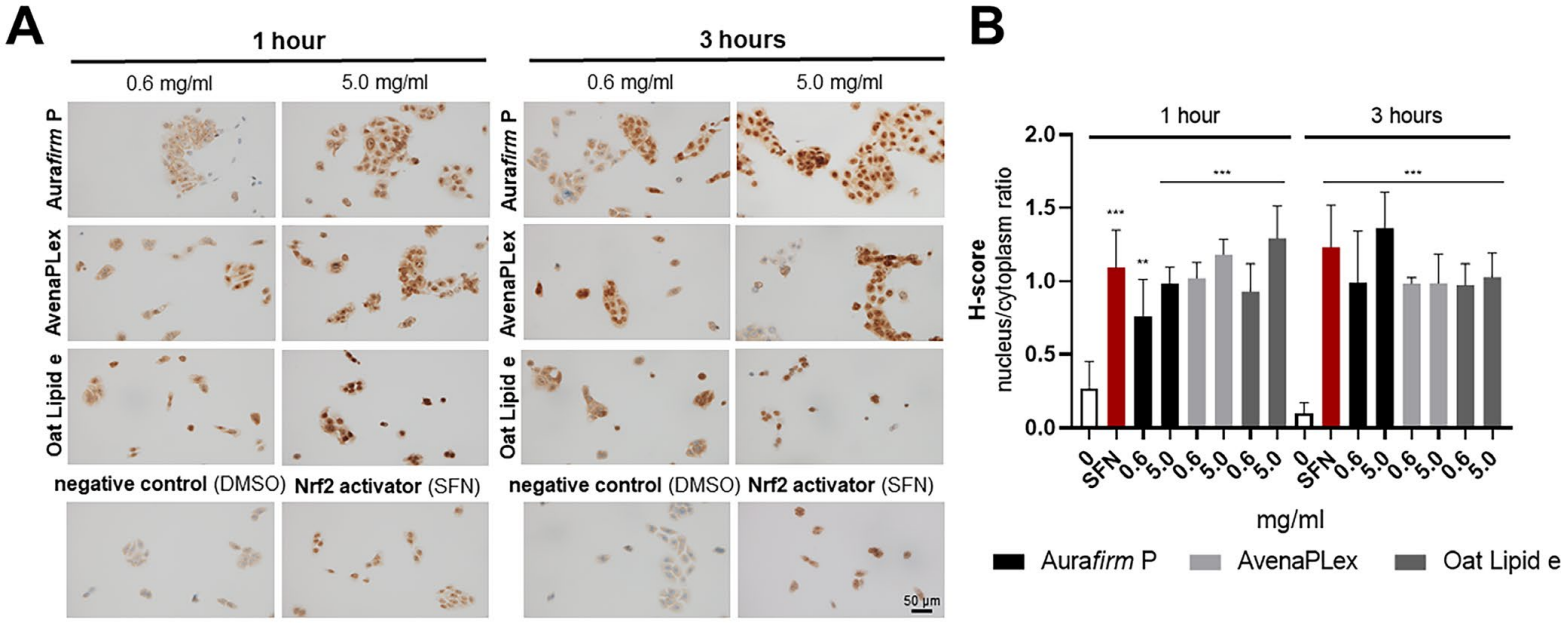
Effect of oat products on subcellular localization of Nrf2 in HaCaT. (A) Cells were treated with oat products (0.6 and 5.0 mg/mL) for 1 or 3 h. Control cells were treated with serum‐free medium containing DMSO (0.5%; *v/v*). SFN (5 μmol/L) was used as a reference compound. The brown color (DAB) represents Nrf2 protein, and the blue color (hematoxylin) a nucleus. Representative microphotographs (magnification 100×) from three independent experiments are shown. Scale bar represents 50 μm. (B) Quantification of Nrf2 intracellular localization is expressed as H‐score (mean ± SD; *n* = 3). SFN represents the reference compound (red column). *p ≤ 0.05, **p ≤ 0.02 or ***p ≤ 0.001 vs. cells treated with DMSO (0.5%; *v/v*).

### 3.7 Effect on AD‐Like Models

AD primarily affects the epidermal layer (keratinocytes), thus only HaCaT cells were used in these experiments. The AD‐like conditions were induced by the mixture of TNF‐α and IFN‐γ (each 10 ng/mL). The chosen concentration of AD inducers did not affect cell viability (Figure 5A,B), but significantly increased the level of both ILs. On average, the level of IL‐6 and IL‐8 were increased 32.9‐ and 9.1‐fold respectively, compared to the control cells (treated with DMSO (0.5%; *v/v*) in serum‐free medium) (Figure 5C–F). The studied oat products were applied either 1 h before (pretreatment; prevention remission model) or 24 h afterwards (posttreatment; therapy model) the application of AD inducers. The efficacy of the oat products was compared with the synthetic glucocorticoid DEX (1 μmol/L; ~0.392 μg/mL) with a potent anti‐inflammatory effect. In the pretreatment model, a nontoxic concentration (0.6 mg/mL) of Aura*firm* P in combination with AD inducers caused a nonsignificant decrease in viability; however, no unfavorable effect was observed at the higher concentration of Aura*firm* P (Figure 5A). In contrast, AvenaPLex at the higher concentration (5.0 mg/mL) caused a significant increase in viability compared to the control. Oat Lipid e had a minimal effect at both concentrations (Figure 5A). With posttreatment (Figure 5B), a significant decrease in viability was found for Aura*firm* P at the lower concentration (0.6 mg/mL), and for Oat Lipid e at the higher concentration (5.0 mg/mL) in combination with AD inducers. No effect of DEX and AD inducers on viability was observed (Figure 5A,B).

**FIGURE 5:**
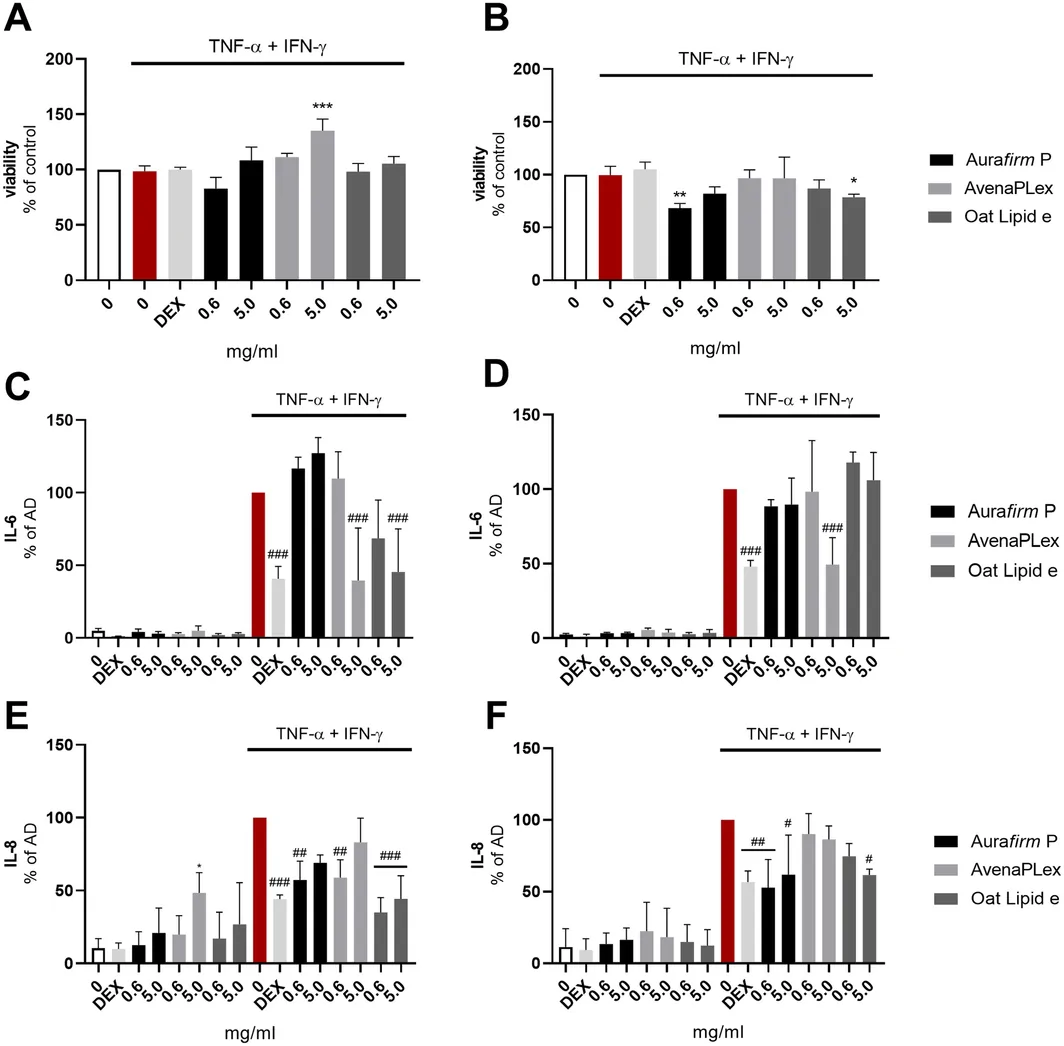
Effect of oat products on cell viability and IL‐6/‐8 levels in AD‐like models. HaCaT cells were treated with oat products (0.6 and 5.0 mg/mL; 1 h) and then TNF‐α and IFN‐γ were applied for 24 h (pretreatment; A, C, E) or TNF‐α and IFN‐γ for 24 h and then oat products (0.6 and 5.0 mg/mL) were applied for 24 h (posttreatment; B, D, F). Control cells were treated with serum‐free medium containing DMSO (0.5%; *v/v*). Cells treated with medium containing TNF‐α and IFN‐γ represent the AD‐like model (red column; 100%). DEX (1 μmol/L) was used as the reference compound. Cell viability was measured by NR uptake and data are expressed as mean ± SD (*n* = 3). *p ≤ 0.05, **p ≤ 0.02 or ***p ≤ 0.001 vs. control cells. The level of ILs was measured by ELISA and data are expressed as % of AD inducer‐treated cells (mean ± SD; *n* = 3). #*p* ≤ 0.05, ##*p* ≤ 0.02 or ###*p* ≤ 0.001 vs. cells treaded with AD inducers. *p ≤ 0.05 vs. control cells.

All the tested oat products and DEX themselves did not stimulate the formation of IL‐6 in HaCaT in both AD‐like models (Figure 5C,D). With IL‐8, the oat products themselves nonsignificantly increased its level compared to control cells, except for AvenaPLex at the higher concentration (5.0 mg/mL) in the pretreatment model (Figure 5E,F).

As expected, the application of DEX significantly decreased the production of IL‐6 and IL‐8 in both experimental AD‐like models (pretreatment and posttreatment), Figure 5C–F. The decrease was down to ~50% of AD inducers treated cells for both ILs and both models. The effect of the studied oat products on the used AD‐like models was different. When oat products were applied before AD inducers (pretreatment; prevention remission model), a concentration‐dependent anti‐inflammatory effect (measured as IL‐6 level) of AvenaPLex and Oat Lipid e was found. At the higher concentration (5.0 mg/mL), their effect was statistically significant and comparable to that of DEX. Aura*firm* P was not effective on this parameter (Figure 5C). However, all three products decreased the level of IL‐8 (Figure 5E). The products were more effective at the lower concentration used (0.6 mg/mL). Oat Lipid e was the most effective of all the tested products, and its effectiveness was comparable to DEX. When the studied oat products were applied after AD inducers (posttreatment; therapy model), only AvenaPLex (5.0 mg/L) significantly decreased the production of IL‐6 with a similar efficacy to DEX. Aura*firm* P also decreased the level of IL‐6, but only insignificantly (Figure 5D). With IL‐8, all products were able to reduce its level. The most pronounced effect was found for Aura*firm* P, whose protection was comparable to DEX at both concentrations used. Oat Lipid e decreased the IL‐8 level in a concentration‐dependent manner. The anti‐inflammatory effect decreased in the order Aura*firm P* > Oat Lipid e >>> AvenaPLex (Figure 5F).

### 3.8 Effect on the NF‐κB Signaling Pathway

The mechanism of the anti‐inflammatory effect of the studied oat products, observed in IL levels in AD‐like models, was tested in transiently transfected Hek‐293 cells with the bi‐directional reporter plasmid pSGNluc. The NF‐κB signaling pathway was activated similarly to in the AD‐like models, by application of TNF‐α and IFN‐γ (each 10 ng/mL). The treatment with MG‐132, an inhibitor of the degradation of IκB, decreased fluorescence in cells treated with AD inducers by about 32.4% ± 1.6%. All three oat products and DEX did not inhibit the activation of the NF‐κB (Figure 6A,B). Therefore, the effect on the level of selected proteins related to NF‐κB signaling was assessed. IκB‐α and phosphorylated p‐NF‐κB p50 were evaluated in the AD‐like model (see above) after the application of AD‐inducers for 30 min (Figure 6C). The inducers decreased the IκB‐α amount and moderately increased the phosphorylation of p50. None of the studied products suppressed the IκB‐α decline. Only a minimal positive effect of DEX on the IκB‐α amount was observed. All studied products alone dose‐dependently decreased IκB‐α level after 30 min of treatment. The effect was concentration‐dependent and statistically significant for AvenaPlex and Oat Lipid e at the higher concentration (5 mg/mL), see Figure 6C. However, IκB‐α amount in oat products treated cells was normalized to the control level within 24 h (Figure S3). All studied products were able to decrease the phosphorylation of p50 and were more effective than DEX. AvenaPLex (5 mg/mL) and Oat Lipid e (0.6 mg/mL) were the most effective. All products without AD inducers moderately reduced p‐p50 level, especially at the higher concentration tested (Figure 6C).

**FIGURE 6:**
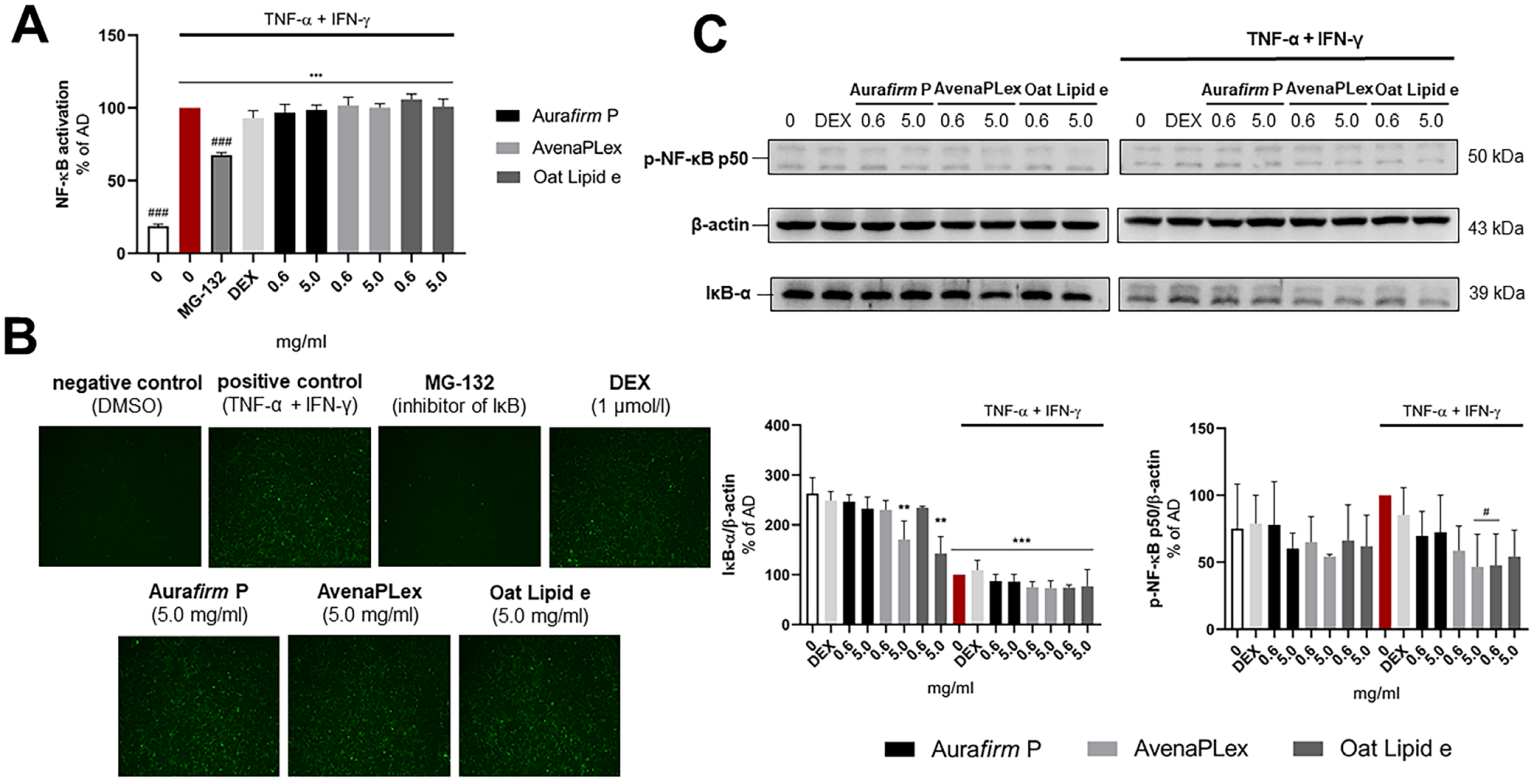
Effect of oat products on activation of NF‐κB signaling pathway. (A) The plasmid pSGNluc‐transfected Hek‐293 cells were preincubated with the studied oat products (0.6 and 5.0 mg/mL), DEX (1 μmol/L) or an inhibitor of IκB degradation (MG‐132; 1 μmol/L) for 1 h and then inflammation was induced with TNF‐α and INF‐γ (each 10 ng/mL). Control cells were treated with serum‐free medium containing DMSO (0.5%; *v/v*). Cells treated with medium containing TNF‐α and IFN‐γ represent the AD‐like model (red column; 100%). The NF‐κB pathway activation is demonstrated as green fluorescence emitted by the EGFP protein. The fluorescence intensity was quantified with the software ImageJ, and results were expressed as % of AD inducer‐treated cells (mean ± SD; *n* = 3). (B) Representative microphotographs, taken at 2.5× magnification, are also shown. ***p ≤ 0.001 vs. control cells; ###*p* ≤ 0.001 vs. cells treated with AD inducers. (C) Levels of IκB‐α and p‐NF‐κB p50 were analyzed in whole‐cell lysates by Western blot. HaCaT cells were preincubated with oat products (0.6 and 5.0 mg/mL) or DEX (1 μmol/L; ~0.39 μg/mL) for 1 h, and then TNF‐α and IFN‐γ (each 10 ng/mL) were applied for 30 min. Control cells were treated with serum‐free medium containing DMSO (0.5%; *v/v*). Cells treated with medium containing TNF‐α and IFN‐γ represent the AD‐like model (red column; 100%). Data were normalized to the reference protein (β‐Actin). Data are expressed as % of AD inducer‐treated cells (mean ± SD; *n* = 3). #*p* ≤ 0.05 vs. cells treated with AD inducers. **p ≤ 0.02 vs. control cells.

### 3.9 Effect on PI3K/Akt Activation

As components of oat were shown to trigger the PI3K/Akt signaling pathway [20], the effect of the studied products on the activation was evaluated. The effect was determined in living Hek‐293 cells transfected with the genetically encoded probe ExRai‐AktAR2. Measured data showed only negligible effects on the direct activation of the Akt enzyme by treatment with oat products (Figure S4).

## 4 Discussion

Recently, a growing consumer preference for natural “green” products has increased the demand for dermal products containing pure‐derived molecules or extracts. Oats are interesting ingredients for dermatological preparations due to their nourishing composition, moisturizing effect and anti‐inflammatory activity [7, 13, 32]. A recent trial showed that after 4 weeks of treatment the oatmeal‐containing topical lotion significantly improved skin symptoms of psoriasis [33]. Aura*firm* P, AvenaPLex, and Oat Lipid e represent commercial oat products that are currently used as sources of oat bioactive components in dermatological preparations at percentage concentrations. For example, Aura*firm* P is integrated into specialized dermocosmetic lines targeting barrier repair and sensitive skin such as Cosmetics à la Carte's Calm Cream [34]. These individual oat products differ in their composition. Aura*firm* P (advanced colloidal oatmeal) is a source of starch, β‐glucans, proteins, amino acids, organic acid and it also contains nonviable *Lactobacillus* bacteria. Therefore, Aura*firm* P is recommended as a prebiotic to stabilize and maintain the skin microbiome and to support epidermal metabolic activity [35]. AvenaPLex contains mainly polar lipids (phospholipids, ceramides), avenanthramides (AVNs) and sterols. In contrast, Oat Lipid e primarily consists of neutral lipids and sterols, with only minimal amounts of phospholipids. Both products also contain natural antioxidants (Table S1). As AvenaPLex and Oat Lipid e represent rich sources of unique lipid complexes, they are utilized to improve/restore lipid barrier function and soothe the skin. Although the favorable effects of these commercial preparations have been described by consumers, their mechanism of action has not been characterized. Thus, this study aimed to assess their biological activities at the cellular level. Our experiments focused on evaluating the products' ability to protect skin cells against extrinsic (UVA radiation) and intrinsic (pro‐inflammatory) stressors, alongside their impact on the related transcription factors, Nrf2 (a stress response) and NF‐κB (an inflammatory response).

UVA radiation (320–400 nm) represents one of the most common environmental stress factors. Its photons penetrate the epidermal and dermal layers, affecting keratinocytes and fibroblasts. The photons stimulate generation of ROS that cause oxidative damage to biomolecules and photoaging [25, 36]. The studied products demonstrated minimal UVA‐protective potential regarding the cell viability of HaCaT and NHDF in both experimental setups (regenerative and photoprotective) (Figure 1A,B). On the other hand, the studied oat products demonstrated a significant ability to reduce intracellular ROS provoked by UVA exposure in keratinocytes in both pretreatment and posttreatment arrangements (Figure 3A,B). A dose‐dependent protection was observed for Aura*firm* P and AvenaPLex in UVA‐exposed keratinocytes. This is crucial not only for mitigating adverse effects of solar radiation such as photoaging, but also for delaying the intrinsic process of skin aging. Our results also indicate that cell viability measurement is not a very sensitive parameter. It provides only preliminary insights into cellular protection. In contrast, the assessment of intracellular level of ROS, the primary consequence of exposure to UVA radiation, has much greater predictive power.

Some pure oat components were reported to have radical scavenging activity [8]. However, all studied products that represent complex mixtures of compounds, exhibited minimal direct antioxidant activity in cell‐free model in a methanol environment (DPPH assay) and in living cell model in an aqueous environment (Fenton‐like reaction) (Figure 2). Consequently, the most significant attribute of the products is likely their indirect antioxidant effect, which was demonstrated at the level of their effect on the Nrf2 intracellular localization [25]. All products significantly stimulated Nrf2 nuclear translocation (Figure 4). It is the first crucial step toward activating of the stress‐response pathway and synthesis of protective proteins (e.g., HO‐1, NQO1, catalase, etc.). This effect on Nrf2 nuclear translocation may be associated with various bioactive compounds found in oat products, such as AVNs, that induce the expression of antioxidant enzymes (e.g., HO‐1, catalase) and protect cells against oxidative damage [17]. Other compounds, for example protocatechuic and syringic acid, have also been reported to activate the Nrf2 pathway and elevate the antioxidant levels (e.g., glutathione, superoxide dismutase, glutathione peroxidase) [37, 38]. As Nrf2 is a key regulator of cellular defense mechanisms (HO‐1, catalase, NQO1, etc.) and simultaneously reduces the production of pro‐inflammatory mediators (IL‐6, IL‐1β) [39, 40], its activation is believed to might be a promising strategy for better managing the symptoms and consequences of AD [41]. The studied products enhanced Nrf2 nuclear translocation, which is essential for the activation of the Nrf2 signaling pathway; however, downstream functional activation (synthesis of specific proteins) was not evaluated and remains a subject for future studies.

AD is a chronic, relapsing dermatosis characterized by persistent itching, leading to thickening and lichenification. Basic therapy aims to reduce dryness and extend symptom‐free periods [23, 42]. The oral and/or topical application of natural substances, including oat, could be used to alleviate the symptoms of AD [43, 44]. In both used AD‐like models in vitro (prevention remission and therapy model), studied products modulated IL‐6 and IL‐8 levels differently than DEX, one of the most commonly used corticosteroids for the short‐term treatment of acute inflammatory skin conditions, including AD. While DEX significantly reduced the level of both ILs (Figure 4C–F) in both models to a similar extent, the oat products modulated IL levels differently. These findings suggest the effect of the products on different phases of AD. IL‐6 has pleiotropic effects, anti‐ and pro‐inflammatory activity, and is involved in both acute and chronic inflammation [45]. IL‐8, a pro‐inflammatory chemokine, affects neutrophils, monocyte maturation, and enhances ROS production [46]. The level of IL‐8 in the *stratum corneum* significantly correlates with the severity of AD [47]. Aura*firm* P significantly decreased IL‐8 in both AD‐like models, especially at the lower concentration, indicating it mainly modulates the IL‐8 controlled pathway. In the pretreatment model, the higher concentration of Oat Lipid e decreased both cytokines, similarly to DEX. However, it showed limited effect in the posttreatment model. This difference may indicate that Oat Lipid e primarily influences cellular processes involved in the response to inflammatory stress when present before inflammatory stimulation. AvenaPLex reduced IL‐6 levels in both AD‐like models, suggesting an ability to modulate inflammation‐associated cellular stress responses.

Since NF‐κB regulates the expression of many pro‐inflammatory molecules, its role was studied in relation to the beneficial effects of oat products on the reduction of ILs in AD‐like models. NF‐κB activation is associated with the degradation of inhibitory proteins (IκB family members), allowing phosphorylation and translocation of the transcription factor p50/p65 into the nucleus [48]. A reporter model (pSGNluc plasmid [29]) was used to determine whether oat products inhibit early steps in the NF‐κB pathway or affect downstream effectors. The results show that the anti‐inflammatory effect of studied products is not associated with the direct effect on the NF‐κB pathway activation, particularly by the blockade of IκB‐α degradation (Figure 6A,B). DEX, a modulator of downstream steps in the pathway, had a negligible effect on NF‐κB activation, which is consistent with its effect described in the literature and confirms the validity of the model [49]. Similarly, none of the studied products protected cells against IκB‐α degradation stimulated by AD‐inducers. Although no effect on NF‐κB activation was observed, we further focused on possible NF‐κB p50 subunit phosphorylation. The products slightly decreased AD‐inducers stimulated p50 phosphorylation (Figure 6C). These results correlate with the inability of the studied products to efficiently prevent IκB‐α degradation and thus NF‐κB activation. However, the pSGNluc reporter system, which determines the transcriptional activity of the NF‐κB protein complex, is unable to detect changes in the phosphorylation status of the p50 transcription factor. p50 subunit phosphorylation is crucial for transcriptional transactivation of specific inflammatory genes [50]. Reduced p50 phosphorylation by studied oat‐based products (Figure 6C) might be a potential underlying mechanism for the downregulation of IL‐6 and IL‐8 (Figure 5). Nevertheless, these findings indicate that oat‐based products have the potential to attenuate the inflammatory response in the other step of complex NF‐κB pathway. This effect may be related to any of oat products components. For example, AVNs were previously found to inhibit IκB‐α degradation and inhibition of transcription factor p65 phosphorylation, IL‐8 release [15], or p50/p65 DNA binding [51]. AVNs have also been reported to stimulate PI3K/Akt activity [16]; however, according to the results obtained, the oat products studied had no effect on the response of the ExRai‐AktAR2 probe that would confirm this effect. All three commercial preparations used in this study are complex mixtures varying in the composition and concentration of individual active oat components. The minimal effect on PI3K/Akt activity may be explained by the fact that content of AVNs in the studied products is probably low to stimulate the response. As shown in the Table S1, in AvenaPLex, the AVNs concentration is low, and for Aura*firm* P and Oat Lipid e is not declared. Based on the results obtained, the studied commercial oat products—Aura*firm* P, AvenaPLex, and Oat Lipid e have ability to attenuate IL‐6 and/or IL‐8 secretion in AD‐like models, most likely through a subtle modulation of downstream NF‐κB cascades rather than upstream IκB‐α protection.

As we demonstrated here, the studied commercial oat products possess the ability to modulate the intracellular localization of the transcription factor Nrf2 (Figure 4). It is evidenced that by controlling the cellular defense, Nrf2 activation downregulates the production of pro‐inflammatory cytokines, such as IL‐6, IL‐8, and TNF‐α [39, 40]. Therefore, the anti‐inflammatory effect of these studied oat ingredients could be mediated via crosstalk between the NF‐κB and Nrf2 signaling pathways. Our results on cellular stress models could support the positive practical experiences that consumers have with products containing oat ingredients.

Several limitations of this pilot study should be noted. All three commercial oat‐based products studied were proved effective across the in vitro stress models used, even though they differ in phytochemical compositions, as is declared by manufacturers' data (Table S1). The exact proportion of the individual components in the used products was not evaluated. A more detailed phytochemical analysis would therefore be appropriate to supplement these findings to characterize the primary active components.

Although these very simple cell‐based systems provided valuable mechanistic insights, these in vitro models (AD‐like and UVA‐stimulated damage) cannot fully reproduce the multicellular complexity, lipid architecture, and systemic immune cell infiltration characteristic of human skin in vivo. In addition, long‐term cumulative effects of oat‐based products could not be evaluated via these simple models due to the inherent lifespan constraints of standard monolayer cell cultures (in vitro systems). It is also important to note a key mechanistic limitation: in this study, the effect of oat‐based products on the Nrf2 signaling pathway was established only on determination of Nrf2 protein nuclear translocation. Downstream functional activation was not studied. Nrf2 translocation is a mandatory prerequisite for the signaling cascade. Therefore, future studies must measure the effect on the expression of target genes (such as HO‐1, NQO1, glutathione synthesizing enzymes, or catalase) via quantitative gene expression and protein analysis.

As mentioned above, the studied oat‐based products are already used as active ingredients in daily skincare products and specialized dermatological formulations [34]. In commercial skincare products, their concentrations range from 1% to 15% (see Table S1). However, at such high, that is, percentage concentrations, it is not possible to apply them directly to the simple cell models used in this pilot study. In intact functional skin, living cells are “protected” by the natural barrier of the outermost layer, the *stratum corneum*, which limits the permeability of substances into the skin. This means that living cells are not directly exposed to the applied amounts of substances, which could be toxic to single‐layer cells. In fact, the compounds penetrate to the deep layers of the skin at much lower concentrations. Therefore, the micromolar concentrations tested in our in vitro models could be sufficient to reflect the actual bioavailable fractions that successfully penetrate the viable epidermis. To confirm capability of the components of studied products to penetrate the skin, it would be very helpful to determine the exact bioavailability and kinetics of transdermal transport using an ex vivo human skin model (Franz diffusion cells). Furthermore, monocultures are grown in an aqueous environment where the solubility of oat‐based products is limited, making it unnecessary to apply such high concentrations.

In conclusion, the studied oat‐based products Aura*firm* P, AvenaPLex, and Oat Lipid e demonstrated significant biological activities in cellular stress models. These findings included promoting antioxidant and anti‐inflammatory properties in vitro. Although these complex oat‐based mixtures differ in their phytochemical profiles, they exhibited a surprisingly comparable efficacy across the used in vitro models. To verify the biological relevance of the above‐mentioned findings, it would be appropriate to carry out further experiments using more complex models, such as human skin explants, in vivo or clinical studies.

## Author Contributions

**Jiří Hanyk:** formal analysis, methodology, investigation, validation and writing – review and editing; **Alena Rajnochová Svobodová:** supervision, validation and writing – review and editing; **Zdeněk Dostál:** methodology and writing – review and editing; **Bohumil Zálešák:** resources and writing – review and editing; **Jitka Vostálová:** conceptualization, formal analysis, methodology, supervision, validation and writing – review and editing.

## Funding

This work was financially supported by grant IGA_LF_2026_009, by the Institutional Support of Palacký University in Olomouc RVO 61989592, and by the Institutional Support of University Hospital in Olomouc RVO 98892.

## Conflicts of Interest

The authors declare no conflicts of interest.

## Supporting information


**Table S1:** Chemical characterisation of oat products available from the website.
**Figure S1:** Effect of oat products on cell viability. HaCaT (A) and NHDF (B) were treated with the oat products (0.6–5.0 mg/mL) for 24 or 48 h. Viability was measured by NR uptake. Cells treated with serum‐free medium with DMSO (0.5%; *v/v*) were used as a control (100%). Results are expressed as the mean ± SD (*n* = 3 for HaCaT; *n* = 6 for NHDF). *p ≤ 0.05 vs. control cells.
**Figure S2:** Effect of oat products on subcellular localization of Nrf2 in HaCaT. Cells were treated with oat products (0.6 and 5.0 mg/mL) for 1 or 3 h. Control cells were treated with serum‐free medium containing DMSO (0.5%; *v/v*). SFN (5 μmol/L) was used as a reference compound (red column). Quantification of Nrf2 intracellular localization (*N* = nucleus; C = cytoplasm) is expressed as H‐score (mean ± SD).
**Figure S3:** Effect of oat products on the IκB‐α‐signaling pathway. Levels of IκB‐α were analyzed in whole‐cell lysates by Western blot. HaCaT cells were pre‐incubated with oat products (0.6 and 5.0 mg/mL) or DEX (1 μmol/L; ~0.39 μg/mL) for 1 h, and then TNF‐α and IFN‐γ (each 10 ng/mL) were applied for 24 h. Control cells were treated with serum‐free medium containing DMSO (0.5%; *v/v*). Cells treated with medium containing TNF‐α and IFN‐γ represent the AD‐like model (red column; 100%). Data are normalized and expressed as % of AD inducer‐treated cells (mean ± SD; *n* = 3).
**Figure S4:** Effect of oat products on Akt activation. The Hek‐293 cells transfected with the genetically encoded probe ExRai‐AktAR2 were perfused with oat products (0.6 mg/mL). The ExRai‐AktAR2 fluorescence intensity was measured over time using cellSens Dimension software. Analyzed cells were chosen randomly based on the expression of the genetically encoded Akt biosensor. For each dataset, three independent experiments were processed (*n* = 3) and the average normalized intensity (mean ± SEM) was plotted. The total number of analyzed cells was as follows: control—15 cells, Aura*firm* P—31 cells, AvenaPLex—32 cells, and Oat Lipid e—31 cells. Representative microphotographs, taken at 40× magnification are also shown. Scale bar represents 2 μm. (0 min—beginning of the experiment, 1 min—after perfusion with HEPES buffer, 8 min—after perfusion with Oat Lipid e (0.6 mg/mL), 12 min—after perfusion with insulin).

## Data Availability

Data will be made available on request.
