# Supplementary material for: Commercial Oat‐Based Cosmetic Ingredients Promote Antioxidant Capacity and Reduce Inflammatory Markers in Skin Cellular Stress Models: An Insight Into Molecular Mechanisms In Vitro
Source: J Cosmet Dermatol. 2026 Oct 14;25(10):e71229. doi: 10.1111/jocd.71229 (PMC13662619; doi:10.1111/jocd.71229)
Supplement: Supplementary file 1 [file JOCD-25-e71229-s001.docx]

**SUPPLEMENTARY MATERIALS AND METHODS**

*Chemicals and reagents*

The three commercial products Aura*firm* P (fermented colloidal oatmeal), AvenaPLex (extracted from crude oat kernel), and Oat Lipid e (oat oil) were provided by the company Oat Services Ltd. (Hampshire, UK).

**Table S1:** Chemical characterisation of oat products available from the website*:

|  | Aura*firm* P |
| --- | --- |
| **recommended usage level**  (skincare applications) | 1-2% |
| dry residue | 20 % |
| starch | 10 % |
| oat beta-glucan | 1 % |
| lipids | *N/A* |
| protein | 3.5 % |
| amino acids | 17,500 mg/kg |
| glutamic acid | 4,000 mg/kg |
| aspartic acid | 1,500 mg/kg |
| leucine | 1,500 mg/kg |
| total organic acids | 4,000 mg/kg |
| lactic acid | 3,000 mg/kg |
| non-viable *Lactobacillus Bacteria* | 200,000 bacterial count/ml |

|  | AvenaPLex | Oat Lipid e |
| --- | --- | --- |
| **recommended usage level**  **(**skincare applications) | 1% | 1-5% |
| polar lipids | 40 % | 6.5-18.5 % |
| ceramides | 4 % | 1-2 % |
| phospholipids | 20 % | 0.5 % |
| mono/digalactosyldiacylglycerols | 15 % | *N/A* |
| other polar lipids | 1 % | 5-16 % |
| neutral lipids | 50 % | 53-65 % |
| triacylglycerols | 37 % | 44-56 % |
| free fatty acids | 8 % | 9 % |
| diacylglycerol | 5 % | *N/A* |
| cholesterol/sterols | 10 % | 9-14 % |
| β-sitosterol | 4 % | *N/A* |
| avenasterol | 4 % | *N/A* |
| other (including cholesterol) | 2 % | *N/A* |
| total avenanthramides | 100 mg/kg | *N/A* |
| tocotrienol & tocopherol | 100 ppm | 300-400 ppm |

*N/A*: not available

*https://oatcosmetics.com/products

Dulbecco’s modified Eagle’s medium – high glucose (DMEM) and heat-inactivated foetal bovine serum (FBS) were obtained from SERANA (Brandenburg, Germany). Trypsin-EDTA (0.25 %), Ham’s F-12 Nutrient Mix, and recombinant mouse epidermal growth factor (EGF) were purchased from Gibco, Lipofectamine 3000 Transfection Kit and the Pierce [BCA Protein](https://www.sciencedirect.com/topics/pharmacology-toxicology-and-pharmaceutical-science/acid-protein) Assay Kit were from Thermo Fisher Scientific (Waltham, MA, USA). PolyJet^TM^ DNA In Vitro Transfection Reagent was from SignaGen Laboratories (Frederick, MD, USA). Stabilized penicillin-streptomycin solution, amphotericin B, neutral red (NR), dimethyl sulfoxide (DMSO), insulin, hydrocortisone, adenine, 3,3',5-triiod-L-thyronin, dexamethasone (DEX), sulforaphane (SFN), 2,2′-azino-bis(3-ethylbenzothiazoline-6-sulfonic acid) diammonium salt (ABTS), bovine serum albumin (BSA), 2,2-diphenyl-1-picrylhydrazyl (DPPH), tocopherol, MG-132 (an inhibitor of IκB degradation), sodium pyruvate (100 mM), dichlorodihydrofluorescein diacetate (H_2_DCFDA), menadione, [ponceau S](https://www.sciencedirect.com/topics/biochemistry-genetics-and-molecular-biology/ponceau-s), [sodium dodecyl sulphate](https://www.sciencedirect.com/topics/pharmacology-toxicology-and-pharmaceutical-science/sodium-dodecyl-sulfate) (SDS), N,N,N′,N′-tetramethylethylenediamine (TEMED), acrylamide-bis-acrylamide mixture (29:1), signal Boost Immunoreaction Enhancer Kit and other common reagents were obtained from Sigma-Aldrich (St. Louis, MO, USA). Ammonium peroxodisulfate was produced by BioChemika Fluka (Czech Republic). [Protease inhibitors](https://www.sciencedirect.com/topics/biochemistry-genetics-and-molecular-biology/protease-inhibitor) Complete tablets were produced by Roche Diagnostics (Germany). Western blotting [luminol](https://www.sciencedirect.com/topics/pharmacology-toxicology-and-pharmaceutical-science/luminol) reagent (ImmunoCruz™) for chemiluminescent horseradish [peroxidase](https://www.sciencedirect.com/topics/biochemistry-genetics-and-molecular-biology/peroxidase) (HRP) detection. [Citric acid](https://www.sciencedirect.com/topics/agricultural-and-biological-sciences/citric-acid), [sodium](https://www.sciencedirect.com/topics/food-science/sodium) citrate, and haematoxylin were produced by Penta Chemicals (Prague, Czech Republic). The immunochemical detection kit Mouse/Rabbit PolyDetector DAB HRP Brown was from Bio SB, Inc. (Santa Barbara, CA, USA). Primary Nrf2 mouse monoclonal IgG (A-10): sc-365949; Primary Nrf2 mouse monoclonal IgG (A-10): sc-365949; primary actin mouse monoclonal IgG (C-2): sc-8432; primary IκB-α rabbit polyclonal IgG (FL): sc-847; primary p-NF-κB p50 mouse monoclonal IgG (A-8): sc-271908; goat anti-rabbit and goat anti-mouse HRP-conjugated secondary antibody was from Santa Cruz Biotechnology, Inc. (Heidelberg, Germany). EnVision FLEX Wash Buffer (20×x), Dako REAL Antibody Diluent and Protein Block Serum-Free were purchased from Dako, Agilent Technologies, Inc. (Santa Clara, CA, USA). Recombinant human tumour necrosis factor-α (TNF-α), recombinant human interferon-γ (IFN-γ), human IL-6 and IL-8 ELISA Development Kits were obtained from PeproTech, Baria (Prague, Czech Republic). pSGNluc was a gift from Baubak Bajoghli & Maria Leptin (Addgene plasmid # 186705; http://n2t.net/addgene:186705; RRID:Addgene_186705). pCS2+HyPer7 was a gift from Vsevolod Belousov (Addgene plasmid # 136466; http://n2t.net/addgene:136466; RRID:Addgene_136466). pcDNA3-ExRai-AktAR2 was a gift from Jin Zhang (Addgene plasmid # 184047; http://n2t.net/addgene:184047; RRID:Addgene_184047).

**SUPPLEMENTARY RESULTS**

**Figure S1:** Effect of oat products on cell viability. HaCaT (A) and NHDF (B) were treated with the oat products (0.6-5.0 mg/ml) for 24 or 48 hours. Viability was measured by NR uptake. Cells treated with serum-free medium with DMSO (0.5 %; *v/v*) were used as a control (100 %). Results are expressed as the mean ± SD (n=3 for HaCaT; n=6 for NHDF). * *p*≤0.05 *vs*. control cells.

**Figure S2:** Effect of oat products on subcellular localization of Nrf2 in HaCaT. Cells were treated with oat products (0.6 and 5.0 mg/ml) for 1 or 3 hours. Control cells were treated with serum-free medium containing DMSO (0.5 %; *v/v*). SFN (5 μmol/l) was used as a reference compound (red column). Quantification of Nrf2 intracellular localization (N = nucleus; C = cytoplasm) is expressed as H-score (mean ± SD).

**Figure S3:** Effect of oat products on the IκB-α-signalling pathway. Levels of IκB-α were analysed in whole-cell lysates by Western blot. HaCaT cells were pre-incubated with oat products (0.6 and 5.0 mg/ml) or DEX (1 μmol/l;~ 0.39 µg/ml) for 1 hour, and then TNF-α and IFN-γ (each 10 ng/ml) were applied for 24 hours. Control cells were treated with serum-free medium containing DMSO (0.5 %; *v/v*). Cells treated with medium containing TNF-α and IFN-γ represent the AD-like model (red column; 100 %). Data are normalized and expressed as % of AD inducer-treated cells (mean ± SD; n=3).

**Figure S4:** Effect of oat products on Akt activation. The Hek-293 cells transfected with the genetically encoded probe ExRai-AktAR2 were perfused with oat products (0.6 mg/ml). The ExRai-AktAR2 fluorescence intensity was measured over time using cellSens Dimension software. Analysed cells were chosen randomly based on the expression of the genetically encoded Akt biosensor. For each dataset, three independent experiments were processed (n=3) and the average normalized intensity (mean ± SEM) was plotted. The total number of analysed cells was as follows: control - 15 cells, Aura*firm* P - 31 cells, AvenaPLex - 32 cells, and Oat Lipid e - 31 cells. Representative microphotographs, taken at 40× magnification are also shown. Scale bar represents 2 μm. (0 min – beginning of the experiment, 1 min – after perfusion with HEPES buffer, 8 min – after perfusion with Oat Lipid e (0.6 mg/ml), 12 min – after perfusion with insulin)
